# Developing genome typing strategies for the emerging zoonotic pathogen *Streptococcus parasuis*

**DOI:** 10.1128/jcm.00741-25

**Published:** 2025-10-21

**Authors:** Xiyan Zhang, Xueli Yi, Wenbo Luo, Jianping Wang, Chaoyuan Yuan, Wenfei Wei, Xuezhen Li, Jinhui Zhang, Han Zheng, Jianguo Xu

**Affiliations:** 1 National Institute for Communicable Disease Control and Prevention, Chinese Center for Disease Control and Prevention 12415, Beijing, China; 2 Center for Medical Laboratory Science, Affiliated Hospital of Youjiang Medical University for Nationalities, Baise, China; 3 Key Laboratory of Research on Clinical Molecular Diagnosis for High Incidence Diseases in Western Guangxi of Guangxi Higher Education Institutions, Baise, China; 4 Baise Agriculture, Animal Husbandry, Fishery Technology Promotion Center, Baise, China; 5 Baise Center for Animal Disease Prevention and Control, Baise, China; 6 Key Laboratory of Coal Environmental Pathogenicity and Prevention, School of Public Health, Shanxi Medical University, Ministry of Education 74648, Taiyuan, China; 7 Research Center for Reverse Microbial Etiology, Workstation of Academician, Shanxi Medical University, Taiyuan, China; 8 National Key Laboratory of Intelligent Tracking and Forecasting for Infectious Diseases, Beijing, China; University of California, Davis, California, USA

**Keywords:** *Streptococcus parasuis*, population, typing, minimum core genome, sequence type

## Abstract

The reported human infections with the emerging zoonotic pathogen *Streptococcus parasuis* are steadily rising. Rapid and standardized genotyping tools specific to *S. parasuis* are critically needed for epidemiological surveillance and identification of strains with zoonotic potential. This study developed a whole-genome sequence (WGS)-based typing strategy, encompassing average nucleotide identity, a minimum core genome (MCG) typing scheme, and a multilocus sequence typing (MLST) scheme using 255 *S*. *parasuis* genomes isolated from eight countries between the 1980s and 2024. The *S. parasuis* population was categorized into 12 MCG clusters based on 72,172 SNPs in non-recombining regions distributed across an MCG comprising 607 genes, forming two distinct lineages. The rapid MCG typing program accurately assigned 92.5% of *S. parasuis* genomes to their corresponding MCG clusters by identifying 4,509 cluster/subcluster-specific SNPs. To elucidate the clonal relationships among *S. parasuis* genomes, an MLST scheme was developed, defining 161 sequence types (STs) based on the allelic profiles of seven housekeeping *loci* (*aroA*, *cpn60*, *gki*, *mutS*, *sdhA*, *recA*, and *thrA*). Thirty-two STs that shared identical alleles at 6 *loci* were assigned to 10 complex clones, whereas 100 STs that shared identical alleles at 4 or more *loci* were grouped into 9 ST clades. The MCG typing scheme and the MLST scheme demonstrated sufficient discriminatory power, with Simpson’s diversity index values of 0.8864 and 0.9821, respectively. This study characterized the *S. parasuis* population and provided a rapid, reproducible, and expandable WGS-based typing strategy for taxonomic identification, epidemiological surveillance, and evaluation of the zoonotic potential of *S. parasuis*.

**IMPORTANCE:**

Our study provides valuable insights for developing effective prevention and control strategies for *Streptococcus parasuis* infections, by revealing the structural characteristics and phylogenetic relationship of *S. parasuis* population, by developing a whole-genome sequence-based typing strategy applicable for epidemiological surveillance, transmission investigation, and zoonotic potential evaluation.

## INTRODUCTION

*Streptococcus parasuis* is an emerging zoonotic pathogen that causes pneumonia, meningitis, peritonitis, pleuritis, and arthritis in humans (1–3). In 2015, *Streptococcus suis* serotypes 20, 22, and 26 were reclassified as *S. parasuis* (4), distinguished from *S. suis* by a specific *recN* gene (5). The first reported human *S. parasuis* infection was identified in the Guangxi Zhuang Autonomous Region (GX), China, in 2021 (2). To date, nine human cases have been reported in China: GX (*n* = 3), Guangdong Province (*n* = 2), Liaoning Province (*n* = 1), Anhui Province (*n* = 1), Sichuan Province (*n* = 1), and Ningxia Hui Autonomous Region (*n* = 1). Due to limitations in taxonomic identification methods, *S. parasuis* is often misidentified as its closely related species *S. suis*, leading in part to an underestimation of its public health significance. A retrospective investigation of human *S. suis* infection cases in Argentina reclassified a 2014 case as *S. parasuis* infection (6). The continuous growth of reported human *S. parasuis* cases underscores the urgent need for epidemiological surveillance and evaluation of its zoonotic potential, necessitating the development of rapid and expandable genomic typing tools. However, molecular typing approaches specific to *S. parasuis* are currently unavailable.

This study analyzed 255 *S*. *parasuis* genomes, representing the broader *S. parasuis* population. The research characterized the population structure and phylogenetic relationships of *S. parasuis* and developed a publicly available whole-genome sequence (WGS)-based identification and typing strategy encompassing average nucleotide identity (ANI), minimum core genome (MCG) typing, and multilocus sequence typing (MLST) scheme. Additionally, the study investigated genetic determinants specific to *S. parasuis* clinical genomes. This research provides valuable insights and applicable genotyping tools for the development of strategies to prevent and control *S. parasuis* infections.

## MATERIALS AND METHODS

### Bacterial strains, identification, and antimicrobial susceptibility

Five strains (A15, A16, A19, A47, and A63) were isolated in 2023 from the throat swabs of healthy pigs in GX. These strains were identified as *S. parasuis* based on their full-length 16S *rRNA* gene sequences and amplification of the *S. parasuis*-specific *recN* gene (5). The strains exhibited >98.7% 16S *rRNA* identity with the *S. parasuis* type strain SUT-286. DNA extraction and sequencing were performed as described in previous studies (7, 8). Additionally, *S. parasuis* genomes from public databases were included in the analysis. SPAdes (v3.15.5) was used for assembly, and FastQC (v0.11.9) and Fastp (v0.23.1) were used for quality control and filtering. Incomplete or gene-redundant *S. parasuis* genomes were excluded. In total, 249 *S*. *parasuis* genomes from NCBI and 1 *S*. *parasuis* genome (GDQY_24_0001SS) from the Chinese Pathogen Identification Net were included (Table S1). ANI analysis of 255 genomes was conducted via http://data.mypathogen.org/pgdb/analysis/task-commit/skani-bacteria. All genomes showed the highest alignment to *S. parasuis* type strain SUT-286, with ANI values ranging from 94.82% to 100.00%. Genome 4253 (9) showed the lowest ANI value (94.82%) but was confirmed as *S. parasuis* through full-length 16S *rRNA*, housekeeping gene analysis, and whole-genome phylogeny (2). Accordingly, 94.82% was established as the ANI threshold for species-level identification. All 255 genomes were thus considered authentic *S. parasuis*, isolated from Myanmar, Japan, China, the United Kingdom, the Netherlands, Switzerland, the United States, and Canada between 1986 and 2024 (Table S1).

The qualities of 255 genomes were evaluated using CheckM2 (v1.1.0) (10). All genomes met the requirements for data analysis, including ≥90% completeness and <5% contamination (Table S2). Additionally, the number of genomes sequenced in this study was assessed using QUAST (v5.2.0) (11), demonstrating an N50 ≥8,000 bp (Table S2).

### Development of the MCG typing scheme

#### Definition of the *S. parasuis* MCG

Gene prediction was conducted using Glimmer (v3.02). OrthoMCL (v2.0.9) was used to identify gene orthologs and construct the genome gene content matrix with a BLAST *E*-value cutoff of 1e^−5^ and an inflation parameter of 1.5. Core genome genes were defined as those present in all genomes. The core genes of 255 *S*. *parasuis* genomes were identified using methods described in our previous study (12). To construct the MCG of the *S. parasuis* population, *tRNA* genes, *rRNA* genes, and mobile genes were excluded from the core genome genes.

#### SNP detection and recombination analysis

ClonalFrameML (v1.13) was used to identify and remove recombined regions in the aligned core genes, which were initially aligned using MUSCLE (v3.8.31). Recombination parameters were estimated using the Baum–Welch Expectation–Maximization algorithm. Gubbins (v3.3.5) was then used to identify SNPs in non-recombined regions.

#### Population structure analysis

Admixture (v1.3.0) was used to infer ancestral subpopulations, assuming 2–15 subpopulations. The optimal *K* value was selected based on minimum cross-validation error in R. Each genome was assigned to the subpopulation with the highest proportion value, with >0.7 considered a reliable assignment. MEGA (v11.0) was used to calculate the *p*-distance (*p* = *n*_d_/*n*) within and between subpopulation clusters, where *n*_d_ represents the number of sites with differences and *n* denotes the total number of sites.

A phylogenetic tree based on core SNPs was generated using the maximum likelihood algorithm and GTRGAMMA model in RaxmlHPC (v8.0.2). Bootstrap analysis was performed with 1,000 replicates. The BS26 genome served as the reference for this analysis. The maximum likelihood inference of the molecular clock phylogeny of the *S. parasuis* population was conducted using TreeTime (V0.11.4) (https://github.com/neherlab/treetime).

#### The MCG rapid typing program

To efficiently assign *S. parasuis* genomes to corresponding MCG clusters, we developed an MCG rapid typing program using an in-house Python script. This program maps the assembled genome FASTA files to the BS26 reference genome and detects MCG cluster/subcluster-specific SNPs. The program calculates the proportion of *S. parasuis* genome assigned to an MCG cluster by dividing the number of matched sites by the number of corresponding cluster/subcluster-specific SNPs. The genome is then assigned to the MCG cluster with the highest proportion value.

### Development of the MLST scheme

#### Housekeeping gene selection

Given the phylogenetic relationship with *S. suis*, seven housekeeping genes (*aroA*, *cpn60*, *dpr*, *gki*, *mutS*, *recA*, and *thrA*) employed in the MLST scheme for *S. suis* (13) were evaluated. All genes were present in the core genome of *S. parasuis*, except *dpr*. Alternative genes, including *ddr* from the MLST scheme of *S. pneumoniae* (14)*, murL* from the MLST scheme of *S. pyogenes* (15)*,* and *sdhA* and *glnA* from the MLST scheme of *S. agalactiae* (16), were considered to replace *dpr*. The *ddr* gene was not part of the MCG of *S. parasuis*. The *d*_N_/*d*_S_ ratios (where *d*_N_ represents nonsynonymous base substitutions and *d*_S_ represents synonymous base substitutions) for housekeeping genes were calculated using paml (v4.10.7). Comparable *d*_N_/*d*_S_ values were observed between *dpr* and *sdhA*. Ultimately, seven housekeeping *loci* (*aroA*, *cpn60*, *gki*, *mutS*, *recA*, *sdhA*, and *thrA*) were selected for the MLST scheme of *S. parasuis* (Table S3).

#### Allele and sequence type (ST) assignment

A new allele was defined as any change in the nucleotide sequence of a housekeeping gene fragment, regardless of alteration of the amino acid sequence. Each genome was assigned a seven-integer number, representing its allelic profile. Genomes sharing identical allelic profiles were designated the same ST. Distinct alleles and STs were numbered based on their distance from the ST1 genome (BS26), calculated using the ETE 3.0 package. The goeBURST algorithm in PHYLOViZ 2.0 (http://www.phyloviz.net/) was used to group STs with one allelic variation into clonal complexes (CCs), while STs with three or fewer allelic variations were grouped into ST clades (SCs). The minimum spanning tree was generated using the MSTree V2 algorithm in GrapeTree (https://github.com/achtman-lab/GrapeTree). CCs and SCs were named after their ancestral STs. The data have been deposited in the PubMLST database, accessible at https://pubmlst.org/organisms/streptococcus-parasuis.

### Discriminatory power of two typing schemes

Simpson’s diversity index (17) was used to evaluate the discriminatory power of two typing schemes (http://www.comparingpartitions.info/index.php?link=Tool), calculated using the formula $Ds=1-\frac{\sum n_{i}(n_{i}-1)}{N(N-1)}$.

## RESULTS

### Development of the MCG typing scheme

In this study, 708 core genes were identified across 255 *S*. *parasuis* genomes, with an average of 0.1684 SNPs/bp. Among these, 101 genes exhibiting high nucleotide diversity (average of 0.3219 SNPs/bp) due to recombination were excluded. Consequently, 607 MCG genes of *S. parasuis* were derived. Comparison with the reference genome of BS26 revealed 78,348 SNPs within the MCG genes. After excluding SNPs in the recombination regions, 72,172 MCG SNP sites in non-recombining regions were identified.

To investigate the population structure of *S. parasuis*, population genetics-based subdivisions were performed, with the optimal CV value of *k* = 12 (Fig. S1). This indicated that the *S. parasuis* population should be divided into 12 ancestral subpopulations, designated as MCG clusters 1 to 12. Based on the MCG cluster with the highest proportion value in a genome, 18, 7, 10, 26, 16, 4, 36, 23, 20, 59, 22, and 14 genomes were assigned to MCG clusters 1 to 12, respectively (Tables S1 and S4). Of the 255 *S*. *parasuis* genomes, 217 genomes were reliably assigned to their corresponding MCG clusters, with the highest proportion value exceeding 0.7. Notably, the proportion value of all genomes assigned to MCG clusters 2, 3, 6, and 10 exceeded 0.7. All *S. parasuis* genomes from patients were unambiguously assigned to MCG cluster 2 (Table S4).

In contrast, 38 genomes were assigned to MCG clusters with the highest proportion value <0.7 and were classified as MCG non-groupable genomes. Based on their highest subpopulation proportion value, 11 genomes were assigned to MCG cluster 7, 7 genomes to MCG cluster 8, 6 genomes to MCG cluster 11, 5 genomes to MCG cluster 12, and 3 genomes to MCG cluster 4. Additionally, MCG clusters 1, 5, and 9 each contained two genomes (Tables S14).

We calculated the genetic distances for all intra- and inter-cluster comparisons. The smallest and largest intra-cluster distances were 0.00005 for MCG cluster 6 and 0.2027 for MCG cluster 5, respectively. The intra-cluster distance is 1.12–4.114 times smaller than the inter-cluster distance. Notably, the intra-cluster distances of MCG clusters 4, 7, and 11 were similar to their mutual inter-cluster distances. Among three MCG clusters, the intra-cluster distance is 1.12–1.32 times smaller than the inter-cluster distance (Table S5).

To examine the phylogenetic relationship among the 12 MCG clusters, a phylogenetic analysis was conducted on the 217 reliably assigned genomes (Fig. S2). The reliably assigned genomes from the same MCG clusters were clustered together in the phylogenetic tree, indicating that the phylogenetic relationship of the 217 genomes aligned with the subpopulation definitions in the structure analysis. The 217 genomes were clustered into two lineages. Lineage 1 consisted of genomes from MCG clusters 3, 6, 9, and 12, while Lineage 2 consisted of genomes from MCG clusters 1, 2, 4, 5, 7, 8, 10, 11, and 12 (Fig. S2). A time-scaled phylogenetic tree was inferred using TreeTime to estimate the emergence timeline of two lineages. The *S. parasuis* population diversified into two lineages before the 1920s, with Lineage 1 emerging earlier (Fig. S3).

**Fig 1:**
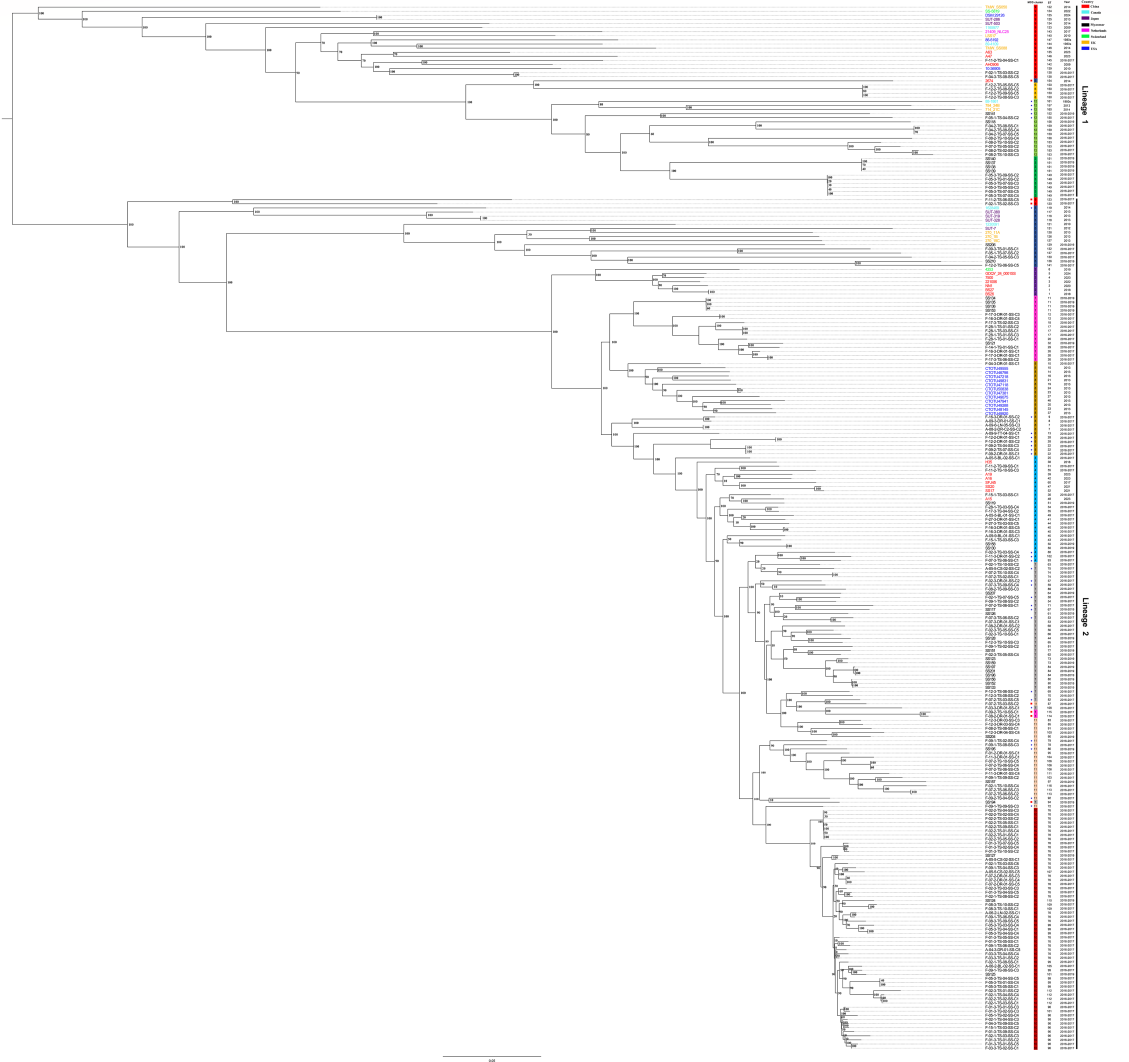
The maximum likelihood phylogenetic tree of 255 *S*. *parasuis* genomes based on core genome SNPs. The genomes were clustered into Lineages 1 and 2. Bootstrap was done with 1,000 replicates. *: The MCG non-groupable genomes clustered with the sub-lineages of their corresponding MCG clusters. ■: The MCG non-groupable genomes not clustered with the sub-lineages of their corresponding MCG clusters. The font color of the genome indicates its country of origin.

To further elucidate the phylogenetic relationship of the 38 MCG non-groupable genomes, a phylogenetic tree comprising all 255 genomes was constructed (Fig. 1). Among the MCG non-groupable genomes, 31 genomes non-reliably assigned to MCG clusters 7 (*n* = 10), 8 (*n* = 7), 11 (*n* = 5), 12 (*n* = 5), 4 (*n* = 3), and 5 (*n* = 1) were clustered into or adjacent to the sub-lineages of their corresponding MCG clusters. However, seven genomes non-reliably assigned to MCG clusters 1 (*n* = 2), 5 (*n* = 1), 7 (*n* = 1), 9 (*n* = 2), and 11 (*n* = 1) were not accurately clustered with corresponding MCG clusters (Fig. 1). Based on the phylogenetic analysis, 248 out of 255 (97.3%) genomes were accurately allocated into their corresponding subpopulations by structure analysis.

To efficiently categorize *S. parasuis* genomes into corresponding MCG clusters, we developed a rapid typing program. For accurate identification of cluster-specific SNPs for each MCG cluster, only the 217 reliably assigned genomes in the structure analysis were used. In total, 3,686 SNPs were determined to be cluster-specific, with 44, 450, 313, 6, 2,797, 4, 10, and 62 specific SNPs for MCG clusters 1, 2, 3, 5, 6, 9, 10, and 12, respectively. No cluster-specific SNPs were identified for MCG clusters 4, 7, 8, and 11. Based on the phylogeny analysis, the MCG cluster 4 was subdivided into seven subclusters, containing 15, 3, 2, 2, 1, 4, and 269 subcluster-specific SNPs, respectively. MCG cluster 7 was subdivided into eight subclusters, containing 10, 41, 4, 4, 6, 4, 5, and 316 subcluster-specific SNPs. MCG cluster 8 was subdivided into two subclusters, containing 5 and 86 subcluster-specific SNPs. MCG cluster 11 was subdivided into three subclusters, containing 2, 31, and 13 subcluster-specific SNPs. The program assigned the genome to the MCG cluster with the highest proportion value, which was calculated by dividing the number of matched sites by the number of corresponding cluster/subcluster-specific SNPs.

In our simulation test, the rapid typing program accurately allocated the 217 reliably assigned genomes into their corresponding MCG clusters with a proportion value of 100% (except for genome 4253, with 99.56%). Among the 38 MCG non-groupable genomes, 10 were allocated to corresponding MCG clusters with proportion values > 0.7, and the results for seven genomes were consistent with those of phylogeny analysis. The remaining 28 MCG non-groupable genomes were assigned to MCG clusters with proportion values < 0.7, and the results for 12 genomes were consistent with phylogeny analysis. Overall, the rapid typing program accurately classified 236 out of 255 (92.5%) genomes into their corresponding MCG clusters. Notably, 98.7% (224 out of 227) of genomes with the highest proportion value > 0.7 were accurately classified into their corresponding MCG clusters.

### Development of the MLST scheme

Seven housekeeping loci (*aroA*, *cpn60*, *gki*, *mutS*, *recA*, *sdh*A, and *thrA*) were selected for the MLST scheme of *S. parasuis*. The *d*_N_*/d*_S_ ratios for all seven *loci* were calculated and found to be substantially less than 0.1, indicating they were not subject to positive selection (Table S3). The number of alleles per locus ranged from 30 to 68, suggesting varying rates of evolution among the seven genes. ST1 was assigned to genome BS26 (Table S1). The number of alleles and STs for the remaining genomes was defined based on their genome phylogenetic distance to genome BS26. A total of 161 STs were identified in the 255 *S*. *parasuis* genomes (Table S6), with 129 STs (80.1%) identified only once (Table S1).

ST76 was the predominant ST, comprising 30 genomes, while the remaining 31 STs contained between 2 and 9 genomes (Table S1). Thirty-two STs (19.9%) were categorized into 10 CCs. CC101 was the largest, containing 53 genomes representing eight STs, followed by CC44, which contained 7 genomes representing 5 STs. CC26 and CC41 contained five genomes representing three STs and four genomes representing four STs, respectively. Each of CC8, CC37, CC50, CC52, CC114, and CC139 contained two genomes representing two STs.

### Relationship between the MCG clusters and STs

The discriminatory power of each typing scheme was evaluated using Simpson’s diversity index, which was 0.8864 (95% CI: 0.8600–0.9100) and 0.9821 (95% CI: 0.9658–0.9984) for the MCG typing scheme and the MLST scheme, respectively. The MLST scheme exhibited higher discrimination power.

In total, 128 STs were assigned to 217 reliably classified genomes in the structure analysis (Table S1). The distribution of STs across MCG clusters was as follows: 8 STs in cluster 1, 6 in cluster 2, 2 in cluster 3, 21 in cluster 4, 13 in cluster 5, 1 in cluster 6, 19 in cluster 7, 15 in cluster 8, 16 in cluster 9, 10 in cluster 10, 13 in cluster 11, and 4 in cluster 12, respectively. The 38 MCG non-groupable genomes comprised 35 STs. Notably, ST53 included both the MCG non-groupable genome F-07-3-DR-01-SS-C1 and the MCG cluster 7 genome F-07-3-TS-06-SS-C2, which were clustered within a sub-lineage in the phylogenetic tree (Table S1; Fig. 1). A similar pattern was observed in CC8, which contained the MCG cluster 8 genome A-09-3-DR-C1-SS-C1 and the MCG non-groupable genome F-16-3-DR-01-SS-C2 (Table S1; Fig. 1).

**Fig 2:**
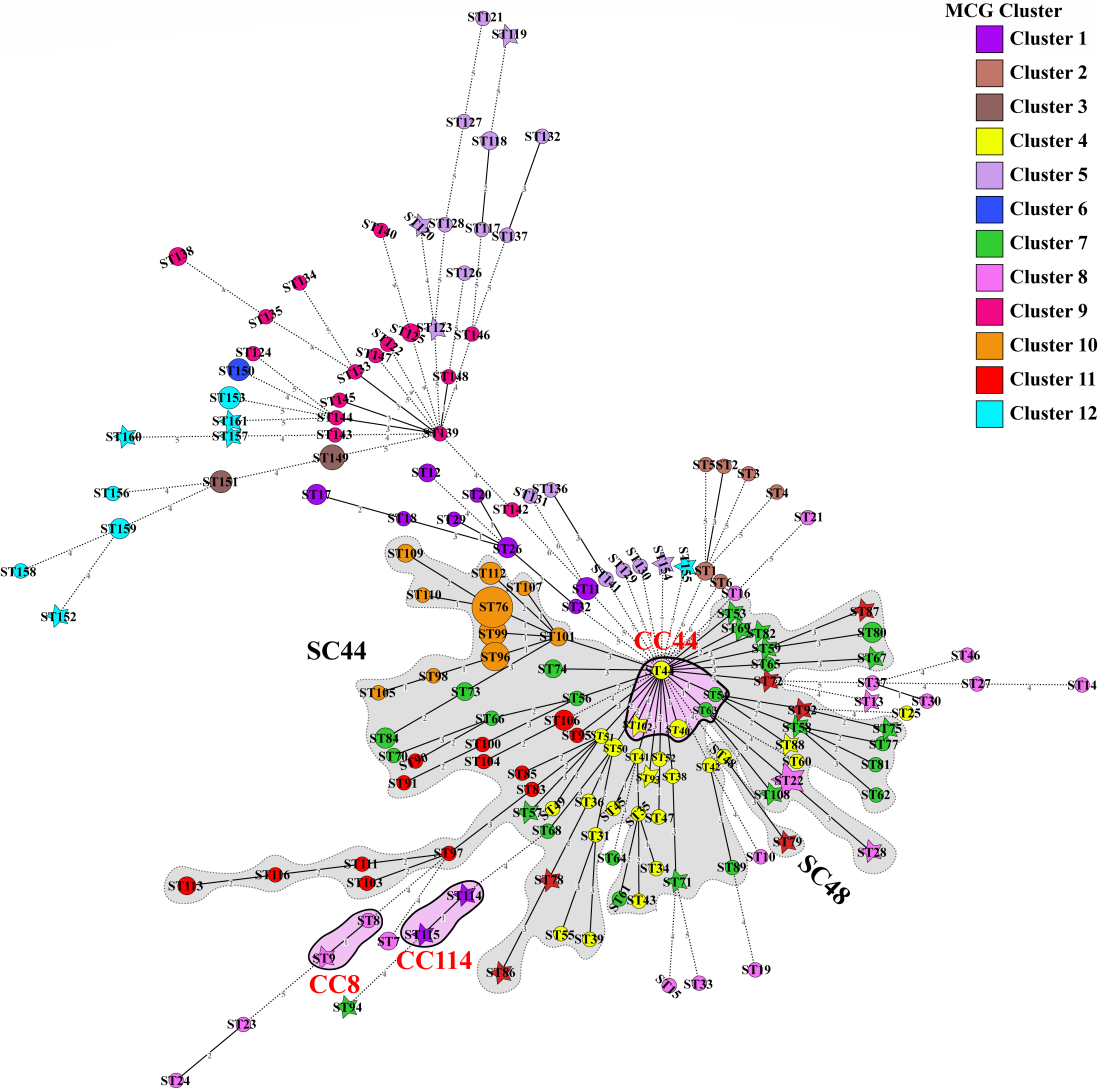
The minimum spanning tree of 161 STs based on the allelic profiles of seven housekeeping *loci*. ◯ designated the ST was assigned to MCG cluster, ☆ designated the ST assigned to MCG non-groupable genome. The size of circle and star denoted the number of genomes assigned to the corresponding ST. Colored zones with full line indicated the STs belong to same CC which contained MCG non-groupable genome; Colored zones with dotted line indicated the STs belong to same SC contained MCG non-groupable genome. The number between two STs represented the number of identical alleles between them.

In the study, the MCG non-groupable genome F-11-3-DR-01-SS-C2 was classified as ST102, which was incorporated into CC44 along with ST44, ST40, ST54, and ST63. Notably, ST44 comprised two genomes, F-27-3-TS-03-SS-C5 and SS128, belonging to MCG clusters 4 and 7, respectively. The two ST40 genomes were classified as MCG cluster 4, while both ST54 and ST63 genomes were categorized in MCG cluster 7 according to the structure analysis (Table S1; Fig. 2). Interestingly, the MCG non-groupable genome F-11-3-DR-01-SS-C2 was non-reliably assigned to MCG cluster 4 in the structure analysis and was positioned adjacent to the sub-lineage of MCG cluster 7 in the phylogenetic tree (Fig. 1 and 2).

To elucidate the phylogenetic relationships of MCG non-groupable genomes, the 100 STs sharing identical alleles at 4 or more *loci* were categorized into 9 SCs, encompassing 24 MCG non-groupable genomes representing 21 STs (Table S1; Fig. 2). SC44 emerged as the largest, comprising 77 STs and 5 CCs, including all genomes from MCG clusters 10 and 11, 20 out of 23 genomes from MCG cluster 4, and 24 out of 25 genomes from MCG cluster 7 (Table S1). This allocation further indicated a close phylogenetic relationship among MCG clusters 4, 7, and 11. Additionally, SC44 contained 23 MCG non-groupable genomes, which were previously non-reliably assigned to MCG clusters 4, 7, 8, and 11. Notably, all genomes non-reliably assigned to MCG clusters 4, 7, and 11 were clustered into SC44, except for the genomes F-09-1-TS-02-SS-C4 (non-reliably assigned to MCG cluster 11) and SS194 (non-reliably assigned to MCG cluster 7) (Table S1; Fig. 2).

### Genomic characteristics of *S. parasuis* genomes from patients

Although six genomes from epidemiologically unrelated sporadic patients were reliably assigned to MCG cluster 2, they exhibited considerable genetic diversity, encompassing five distinct STs (Table S1). Moreover, the five STs shared no more than five alleles with each other. This observation underscores the significant genetic diversity among *S. parasuis* clinical genomes.

A specific gene cluster of 1,270 bp containing three genes unique to *S. parasuis* clinical genomes was identified. The three genes, transcribed in the same direction, are as follows: the first gene, *BS26-JOA01_09175* (nucleotides 1–123), encodes a 40-amino acid protein exhibiting complete homology (100% identity) to the 5′ side of RNase adapter RapZ (296 amino acids) found in *Streptococcus vestibularis*. The second gene, *BS26-JOA01_09180* (nucleotides 104–874), encodes a 256-amino acid protein showing high homology (95% identity) to a cyclic nucleotide-binding protein in *S. thermophilus*. The third gene, *BS26-JOA01_09185* (nucleotides 1,082–1,270), encodes a 62-amino acid protein with substantial homology (94% identity) to a serine protease in *S. thermophilus*.

## DISCUSSION

*S. parasuis* is an emerging zoonotic pathogen (2), and its public health threat is urgently needed to be thoroughly evaluated. The infection caused by the emerging zoonotic pathogen *S. parasuis* is associated with the interdependence of human, animal, and environmental dimensions. The 255 *S. parasuis* genomes analyzed in this study were isolated from the environment, healthy pigs, diseased pigs, a diseased calf, and human patients. To effectively control and prevent human *S. parasuis* infection, a One Health approach is necessary to analyze the epidemiological characteristics, transmission patterns, and evolutionary relationships of strains isolated from the environment and animals.

Due to the lack of specific taxonomic identification methods at the genome level, *S. parasuis* has often been misidentified as *S. suis*, leading in part to an underestimation of its public health importance. In our previous study, 241 genomes from divergent populations of “*S. suis*” (18) were reclassified as *S. parasuis* using pairwise ANI comparisons (19). There is an urgent need to develop WGS-based schemes to identify and genotype *S. parasuis* for epidemiological surveillance and pathogenic strain discrimination. ANI is widely used to classify bacterial species by comparing the similarity between query genomes and type/reference genomes of corresponding species (20), with values ranging from 93% to 96% typically used as the threshold for species identification (21). Recently, ANI has been used to define intra-species units, such as circumscription of strain, STs, and CCs (22–25). In our previous study, the ANI value for *S. suis* species definition was calculated as 93.17% based on pairwise ANI comparisons between the type genome and 2,422 genomes from the central group of *S. suis* (19). In the present study, an ANI threshold value of 94.82% was determined for classifying *S. parasuis* species through pairwise ANI comparisons with the type genome SUT-286. Accurate identification of *S. parasuis* species provides the foundation for subsequent population structure analysis and the establishment of a WGS typing strategy.

The MCG, defined as the non-mobile genes present in all strains of a species, was proposed in our previous study (12). The SNP sites with a high frequency of recombination were removed from the MCG genes. The structural analysis based on the SNPs in non-recombining regions of MCG in combination with phylogenetic analysis divided the species population into optimal MCG clusters. These clusters may serve as taxonomic units to identify the MCG clusters with the potential to cause human infections and outbreaks (12). The MCG typing scheme has already been widely adopted in genomic epidemiology investigations of *S. suis* (7, 26–30). In the present study, a similar approach was also employed to analyze the population structure and divide the ancestral subpopulation of *S. parasuis*.

The study divided the *S. parasuis* population into 12 subpopulations, which were grouped into two distantly related lineages. The time-scaled phylogeny revealed that the rapid population expansion of *S. parasuis* occurred after the 1960s, consistent with observations for the *S. suis* population and closely associated with the widescale introduction of indoor rearing of meat-producing pigs (31). The results of both structure and phylogeny analyses were highly concordant, although 38 genomes assigned to corresponding MCG clusters were deemed unreliable in the structure analysis. It is possible that these MCG non-groupable genomes located in the evolutionary intermediate state possessed mixed phylogenetic signals of two MCG clusters, which inferred their subpopulation assignments. The significant unidentified recombinant history of MCG non-groupable genomes might also be responsible for the discrepancy between the structure and phylogeny analyses. Novel strategies are required to identify the recombinant SNPs and improve the reliability of structure analysis.

In this study, we developed the MCG rapid typing program to replace the complex and time-consuming structure analysis. The program offers an interim method for MCG typing in laboratories where computational resources and bioinformatics expertise are limited. Although no cluster-specific SNPs for MCG clusters 4, 7, 8, and 11 were identified, over 92% of genomes were accurately assigned to corresponding MCG clusters by the rapid typing program, with the accuracy approaching that of the structure analysis. With more genomes from MCG clusters 4, 7, 8, and 11 analyzed and greater interferences from recombinant SNPs eliminated, the subcluster-specific SNPs are expected to be replaced by cluster-specific SNPs to improve the accuracy of the rapid typing program in future studies.

MLST is crucial for elucidating the evolutionary characteristics and relationships of individual genomes. To date, MLST typing schemes, considered the gold standard for epidemiological research, have been developed for 141 species, including 14 species within the *Streptococcus* genus (https://pubmlst.org/). The MLST scheme for *S. suis* has been widely applied in epidemiological and pathogenic studies (32–36). However, the primers of the MLST scheme for *S. suis* cannot be applied to amplify *S. parasuis* strains due to significant differences in the sequence of housekeeping *loci* between the two species. Moreover, the *dpr* gene is not among the MCG genes of *S. parasuis*. In this study, a WGS-based MLST scheme for *S. parasuis* was developed by retaining six housekeeping gene *loci* from *S. suis* and replacing the *dpr* gene with the *sdhA* gene. In the study, some genomes with the same STs were not clustered together in the phylogenetic tree. Similar polyphyletic ST25 (37), ST373 (7, 38), ST1 (39), and ST7 (34) were also observed in *S. suis*. In addition, the non-monophyletic pattern of some STs may indicate the inaccurate genotyping by only using the MLST scheme, which was found in the important human pathogen *Acinetobacter baumannii* (40), due to high genetic variation (41) and substantial levels of recombination (42). The findings highlight that the MLST might produce inaccurate genotyping for some pathogens and the necessity of combining genotyping strategies based on whole genomes to accurately reveal the relationships among *S. parasuis* genomes. The MCG sequence typing scheme can be used to classify *S. parasuis* genomes at the subpopulation level, while the MLST scheme helps distinguish the evolutionary differences among genomes from the same MCG cluster. Furthermore, our data indicated that the MLST scheme could provide valuable insights into the evolutionary relationships of MCG non-groupable genomes, enhancing our understanding of the phylogenetic relationships of MCG non-groupable genomes, particularly those non-reliably assigned to MCG clusters 4, 7, and 11. The combination of MLST and MCG typing schemes provides strong discriminatory power to reveal the phylogenetic relationships and evolutionary linkages of *S. parasuis* genomes.

In this study, all genomes from human *S. parasuis* infection cases were unequivocally assigned to MCG cluster 2. The genomes from patients were also confined to specific and distinct STs. The MCG typing and MLST schemes provide valuable clinical and public health information necessary for precise prevention and control of human *S. parasuis* infections. Furthermore, a gene cluster containing three genes was identified as specific to clinical genomes. Notably, the gene encoding the partial adaptor protein RapZ was identified as a putative pseudogene, which is involved in regulating sRNA degradation (43). Further research is required to investigate the function of this cluster and determine whether these genetic determinants could be used to distinguish strains with the potential to cause human infections.

Most of the genomes used in the study were isolated from Myanmar between 2016 and 2019. A limitation of this study is the genomic bias in geographic and temporal distribution. Although considerable genomic diversity was revealed among the 255 genomes, adding new *S. parasuis* strains is necessary to expand the *S. parasuis* population and enhance the scheme’s discriminatory power for *S. parasuis* epidemiological surveillance.

In conclusion, this study revealed the high heterogeneity of the *S. parasuis* population. A WGS-based typing strategy, encompassing an MCG typing scheme and MLST scheme, was developed. This strategy translated the high-throughput sequencing data into accurate, reproducible, expandable, and comparable genotyping data. The resulting data are applicable for surveillance of epidemiology, investigation of evolutionary relationships, and identification of strains with zoonotic potential. Our study provides valuable insights for developing effective prevention and control strategies for *S. parasuis* infections.

## Data Availability

The five newly sequenced genomes and genome GDQY_24_0001SS have been deposited in the NCBI database under BioSample accessions SAMN47559535–SAMN47559540. The MCG rapid typing program is accessible at https://github.com/1111zxy/Streptococcus_parasuis_MCG.py.

## AFTER EPUB

*[This article was published on 21 October 2025 with an error in Jianguo Xu’s given name. The name was corrected in the current version, posted on 28 October 2025.]*

## SUPPLEMENTAL MATERIAL

Figure S1 jcm.00741-25-s0001.tif The Cross-Validation error value of K value assuming 2 to 15. K= 12 with minimum Cross-Validation error value was optimal.

Figure S2 jcm.00741-25-s0002.tif The Maximum Likelihood phylogenetic tree of 217 *S. parasuis* genomes based on core genome SNPs. The genomes were clustered into Lineage 1 and Lineage 2. Bootstrap was done with 1000 replicates. The font color of the genome indicates its country of origin.

Figure S3 jcm.00741-25-s0003.tif The time-scaled phylogeny of the *S. parasuis* population. TreeTime was used to transform the maximum likelihood phylogenetic tree of 217 reliably assigned *S. parasuis* genomes into a dated tree.

Table S1 jcm.00741-25-s0004.xlsx The information of *S. parasuis* genomes used in the study.

Table S2 jcm.00741-25-s0005.xlsx The quality of 255 genomes used in the study.

Table S3 jcm.00741-25-s0006.xlsx The characteristics of housekeeping loci used in the MLST scheme for *S. parasuis*.

Table S4 jcm.00741-25-s0007.xlsx The proportion value of 255 *S. parasuis* genomes in the structure analysis.

Table S5 jcm.00741-25-s0008.xlsx The genetic distance for 12 MCG cluster.

Table S6 jcm.00741-25-s0009.xlsx The allelic profiles of 161 STs for *S. parasuis*.

ASM does not own the copyrights to Supplemental Material that may be linked to, or accessed through, an article. The authors have granted ASM a non-exclusive, world-wide license to publish the Supplemental Material files. Please contact the corresponding author directly for reuse.
