# Supplementary figures and images for: Developing genome typing strategies for the emerging zoonotic pathogen Streptococcus parasuis
Source: J Clin Microbiol. 2025 Oct 21;63(11):e00741-25. doi: 10.1128/jcm.00741-25 (PMC12607890; doi:10.1128/jcm.00741-25)

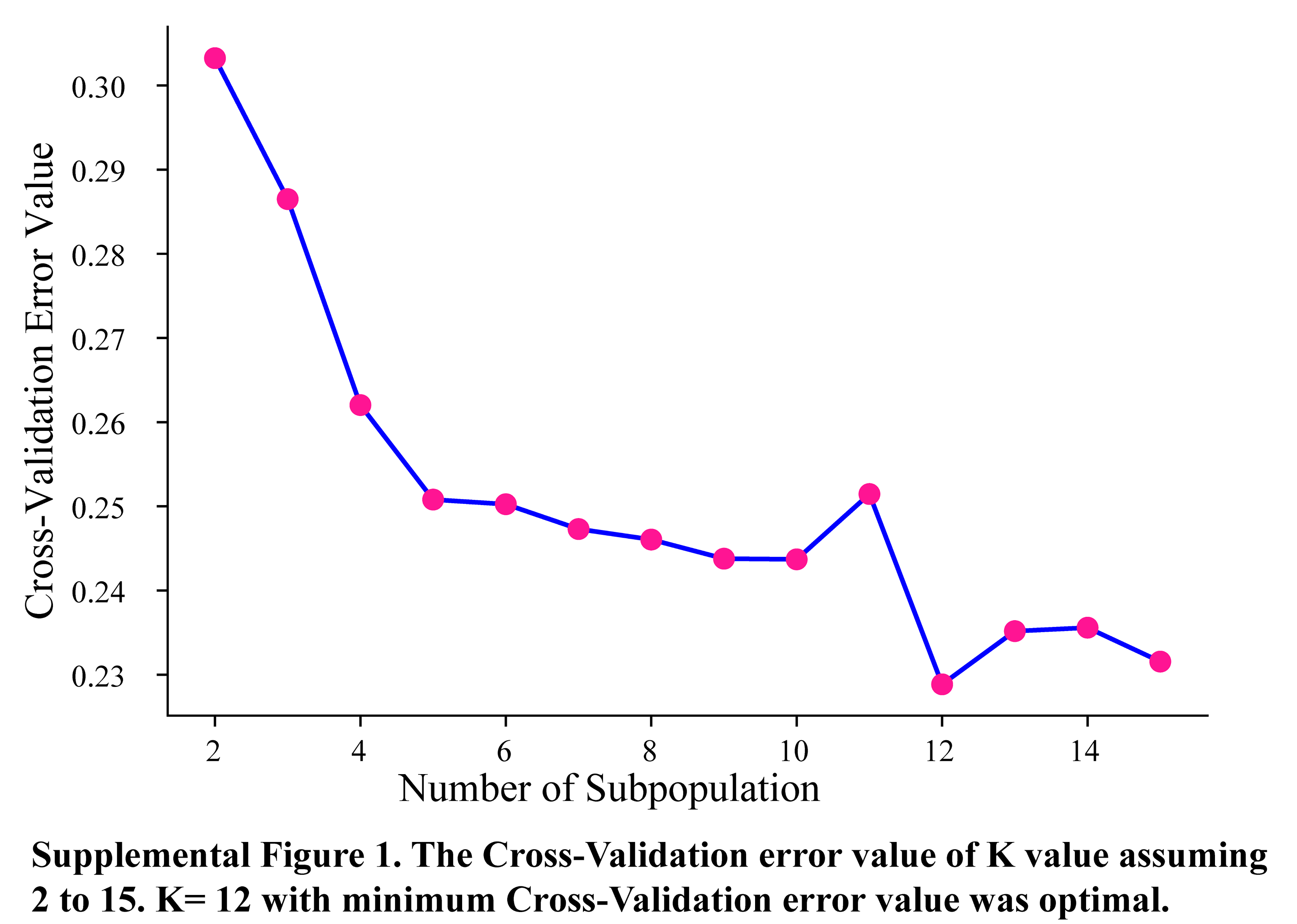

Supplement: Figure S1 — The Cross-Validation error value of K value assuming 2 to 15. K= 12 with minimum Cross-Validation error value was optimal. [file jcm.00741-25-s0001.tif]

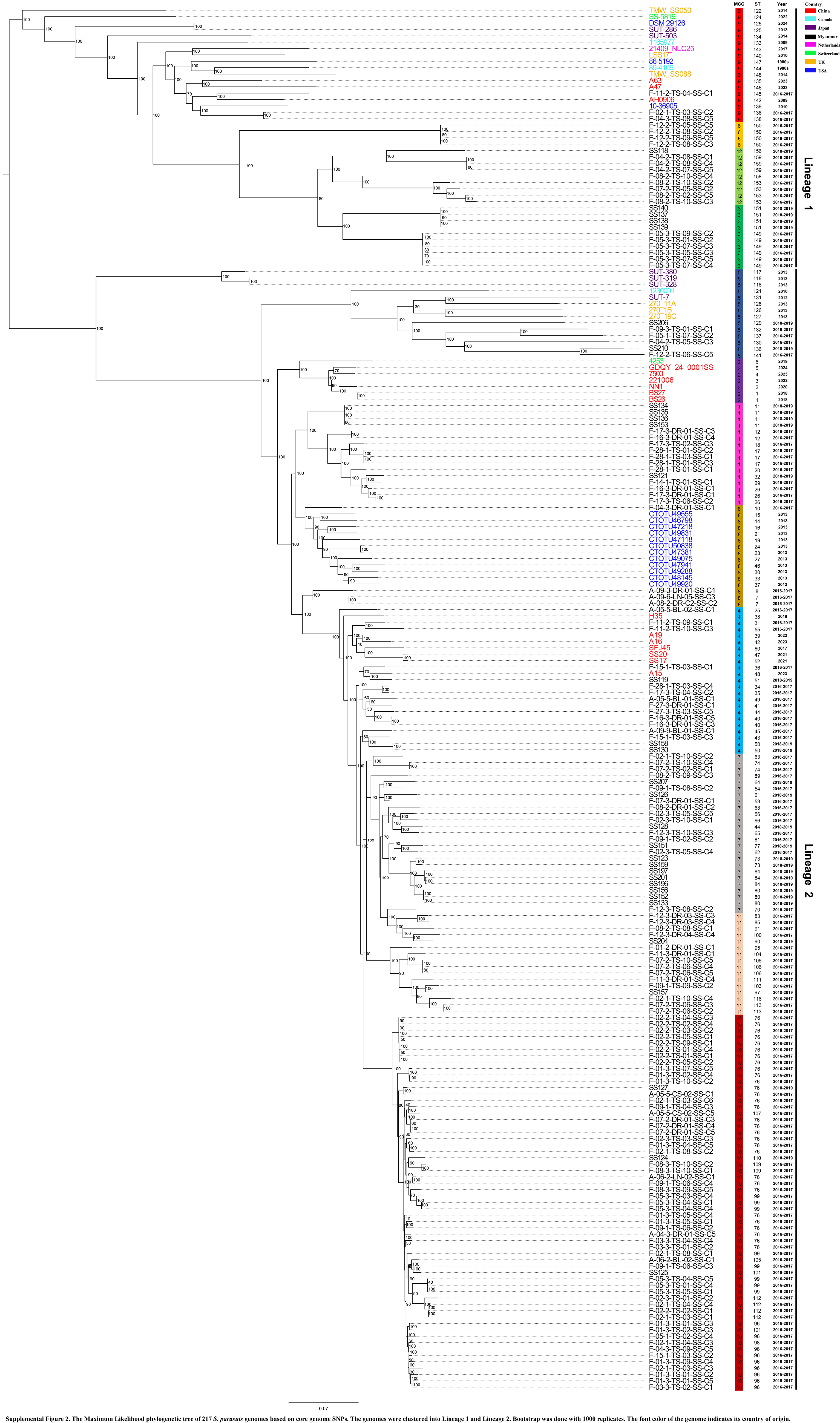

Supplement: Figure S2 — The Maximum Likelihood phylogenetic tree of 217 S. parasuis genomes based on core genome SNPs. The genomes were clustered into Lineage 1 and Lineage 2. Bootstrap was done with 1000 replicates. The font color of the genome indicates its country of origin. [file jcm.00741-25-s0002.tif]

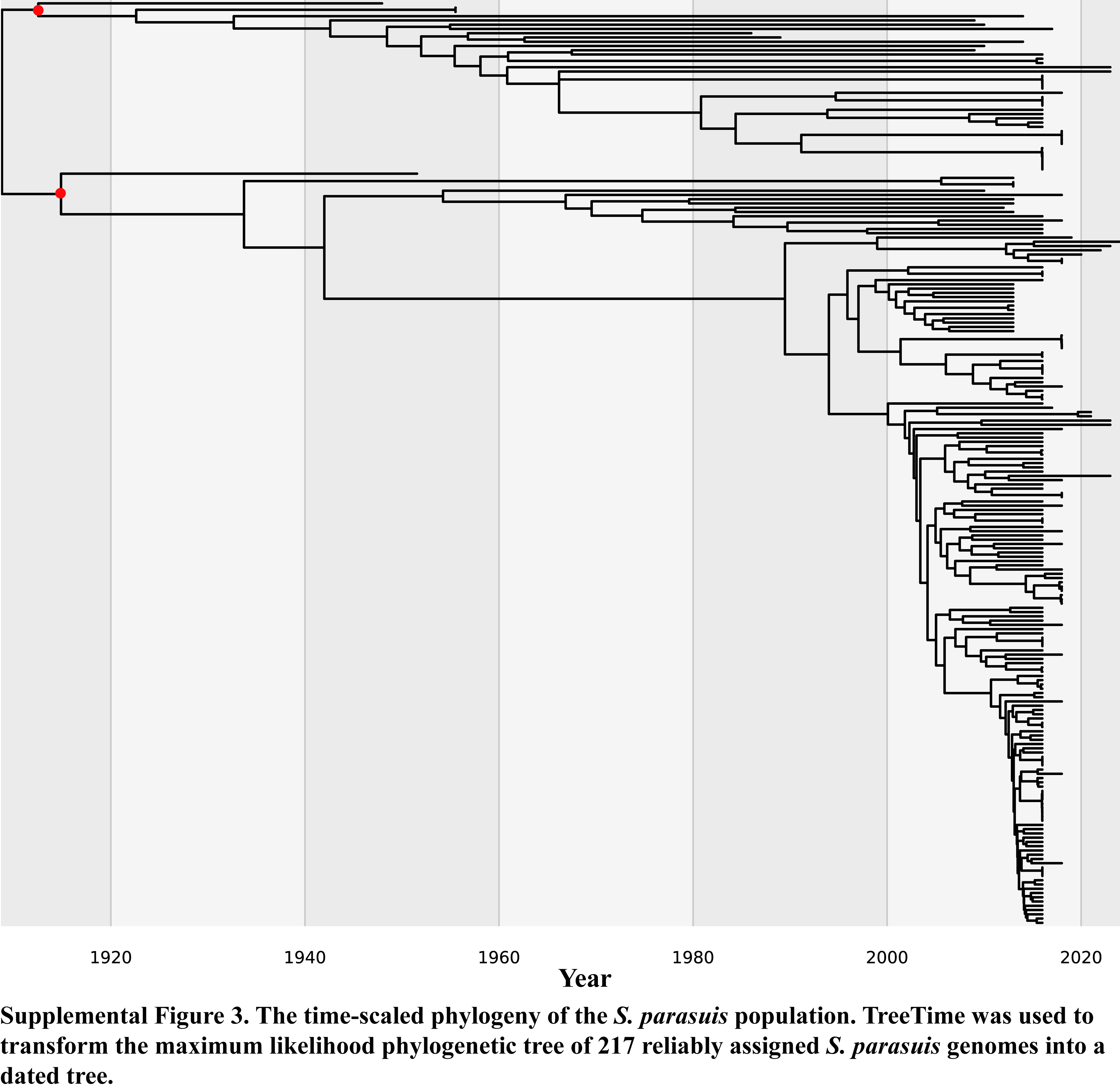

Supplement: Figure S3 — The time-scaled phylogeny of the S. parasuis population. TreeTime was used to transform the maximum likelihood phylogenetic tree of 217 reliably assigned S. parasuis genomes into a dated tree. [file jcm.00741-25-s0003.tif]
